# Evolutionary trajectory of bacterial resistance to antibiotics and antimicrobial peptides in *Escherichia coli*

**DOI:** 10.1128/msystems.01700-24

**Published:** 2025-02-27

**Authors:** Feiyu Yu, Dejuan Wang, Haijie Zhang, Zhiqiang Wang, Yuan Liu

**Affiliations:** 1 Jiangsu Co-innovation Center for Prevention and Control of Important Animal Infectious Diseases and Zoonoses, College of Veterinary Medicine, Yangzhou University, Yangzhou, China; 2 Joint International Research Laboratory of Agriculture and Agri-Product Safety, The Ministry of Education of China, Yangzhou University, Yangzhou, China; 3 Institute of Comparative Medicine, Yangzhou University 38043 , Yangzhou, China; Zhejiang University College of Animal Sciences, HangZhou, Zhejiang, China

**Keywords:** antibiotics, antimicrobial resistance, antimicrobial peptide, fitness cost, collateral sensitivity

## Abstract

The global crisis of antimicrobial resistance poses a major threat to human health, underscoring the urgency of developing new antibacterial strategies. Antimicrobial peptides (AMPs) are promising alternatives to antibiotic therapy, yet potential microbial resistance is of great concern. Resistance is often accompanied by fitness costs, which may in turn influence the spread of drug-resistant bacteria and their susceptibility to other antimicrobial agents. Herein, we investigate the evolutionary trajectory of bacterial resistance to antibiotics and AMPs in *Escherichia coli*, and evaluate the fitness costs and collateral sensitivity of drug-resistant strains. We reveal that *E. coli* develops resistance to antibiotics, particularly ciprofloxacin and kanamycin, at a notably faster rate than to AMPs. Moreover, antibiotic-evolved strains exhibit slightly higher fitness costs than AMP-evolved bacteria, primarily manifested in reduced bacterial growth and swimming motility. Notably, we demonstrate that trimethoprim-resistant *E. coli,* with mutations in *thyA* gene, displays enhanced susceptibility to pexiganan, as evidenced by both *in vitro* and *in vivo* studies. Overall, our findings shed new insights for the clinical deployment of AMPs and propose innovative therapeutic strategies for combating antibiotic-resistant bacterial infections.

**IMPORTANCE:**

The global spread of antimicrobial resistance necessitates the development of innovative anti-infective strategies. Antimicrobial peptides (AMPs) represent promising alternatives in the post-antibiotic era. By monitoring the evolutionary trajectory of bacterial resistance to eight antibiotics and ten AMPs in *Escherichia coli*, we demonstrate that *E. coli* exhibits slower emergence of resistance against AMPs compared with antibiotics. Additionally, these antibiotic-resistant strains incur significant fitness costs, particularly in bacterial growth and motility. Most importantly, we find that some antibiotic-resistant strains show collateral sensitivity to specific AMPs in both *in vitro* and animal infection models, which is conducive to accelerating the development of AMP-based antibacterial treatment.

## INTRODUCTION

The rapid emergence and dissemination of antibiotic-resistant bacteria represents an escalating threat to global public health, necessitating the urgent development of new antimicrobial strategies. Antimicrobial peptides (AMPs), a class of polypeptides widely distributed in nature, is a vital component of the innate immune system (1, 2). Characterized by their low molecular weight and minimal secondary structure, AMPs can adopt a variety of conformations, such as α-helices and β-sheets. Most AMPs, typically ranging from 5 to 50 amino acids in length, lack enzymatic activity and can function in both monomeric and polymeric conformations (3). Nowadays, AMPs have emerged as promising antibiotic alternatives with broad-spectrum efficacy against bacteria and fungi (4, 5). Unlike conventional antibiotics, which target specific processes, such as cell membrane destruction, DNA replication, or protein synthesis (6), some AMPs have multiple modes of action on non-specific targets (7), thereby reducing the likelihood of drug resistance (8). AMPs exert their antimicrobial effects through two primary mechanisms as follows: membrane-targeting AMPs disrupt the structural integrity of bacterial cell membranes, while non-membrane-targeted AMPs inhibit the synthesis of nucleic acids, enzymes, and other functional proteins (9). These features make AMPs less susceptible to microbial resistance. However, resistance to AMPs has also been reported in recent years, including mechanisms such as capturing and inactivating AMPs, cleaving AMPs with proteases, altering bacterial surface charge to reduce affinity and inducing specific gene expression (10). Despite these possible resistance mechanisms, AMPs are still promising anti-infective agents due to their unique mechanisms of action and broad-spectrum antimicrobial activity. For example, a recent study identified a potential AMP encoded by a small open reading frame named prevotellin-2 from the human microbiome, demonstrating antimicrobial activity comparable to that of polymyxin B (11). Although AMPs face hurdles in clinical application, such as potential toxicity to humans and instability under specific environmental conditions, their unique mechanisms of action offer new possibilities for anti-infection therapy.

The acquisition of resistance is usually accompanied by a fitness cost, manifesting as reduced viability, transfer rate, and virulence of resistant strains compared to wild-type strains in the absence of antibiotics (12, 13). This fitness cost is associated with resistance mutations that target critical biological functions. For example, fluoroquinolone resistance in *Pseudomonas aeruginosa* impairs motor ability (14), while aminoglycoside resistance alters ribosome structure (15, 16), thereby interfering with essential cellular functions. The evolution of antibiotic resistance allows bacteria to survive antibiotic pressure but also imposes a genetic burden. As antibiotic pressure is relieved, resistance mutations can weaken normal physiological and biochemical activities, adversely affecting bacterial survival (17).

Beyond developing new antibiotics, innovative therapeutic strategies are essential to combat the antibiotic resistance crisis. Recently, collateral sensitivity-based therapy has garnered significant attention. Collateral sensitivity refers to the phenomenon where bacteria resistant to one antibiotic or a specific class of antibiotics become more sensitive to others (18). This negative cross-resistance is a common pleiotropic consequence of drug-resistance gene mutations (19, 20) and is frequently observed between antibiotics with different antimicrobial mechanisms. For instance, the increased resistance to aminoglycoside antibiotics in *Escherichia coli* is often coupled with increased susceptibility to β-lactam antibiotics (21, 22). This phenomenon has inspired a novel collateral sensitivity-based therapeutic approach, which not only reverses the reduced efficacy of drugs but also effectively eliminates pathogenic bacteria. It has been proposed that collateral sensitivity could reduce or prevent the emergence of multidrug resistance, and the potential utility of sequential or combination therapy has been supported by experimental studies on several bacterial species (20, 23, 24). However, there is still a lack of systematic research on the evolution and fitness costs of antibiotic and AMP resistance, as well as the possible collateral sensitivity.

In this study, we investigated the patterns and rules of drug resistance evolution in ten AMPs and eight antibiotics through experimental evolution under sub-inhibitory concentrations. Moreover, we comprehensively compared the fitness costs associated with resistance to these two classes of antimicrobial substances and identified effective drug pairs with collateral sensitivity. Our findings provide a theoretical foundation for guiding the clinical application of AMPs and for developing new treatment options against antibiotic-resistant bacterial infections.

## RESULTS

### Experimental evolution of bacterial resistance to antibiotics and AMPs

**Fig 1:**
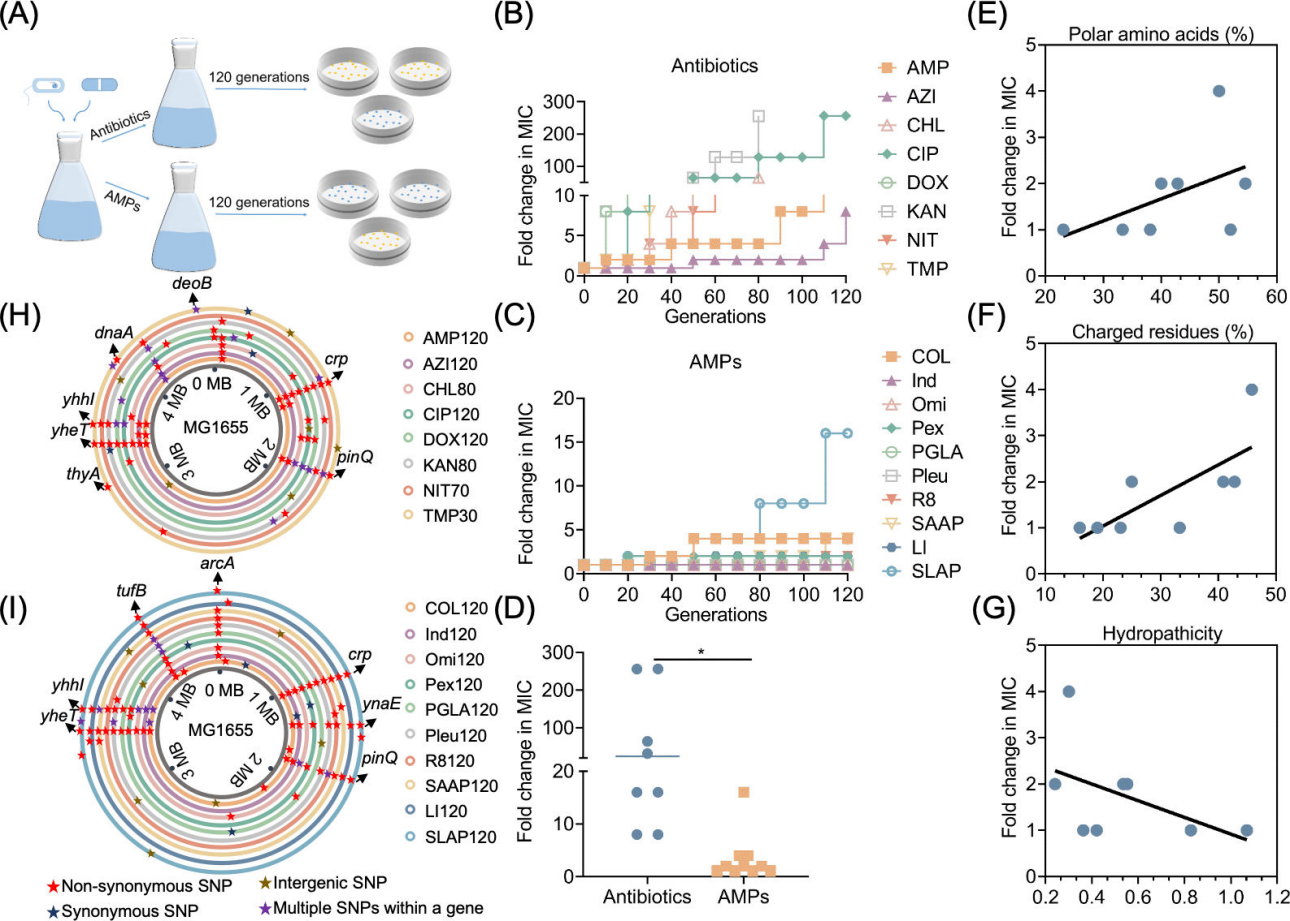
Evolutionary analysis of bacterial resistance to antibiotics and AMPs in *E. coli*. (**A**) Schematic diagram of the pattern of experimental evolution *in vitro*. (**B**) Dynamics of stepwise resistance development to different antibiotics in MG1655. Each point represents the changes in MIC values against different antibiotics. (**C**) Dynamics of stepwise resistance development to different AMPs in MG1655. Each point represents the changes in MIC values against different AMPs. (**D**) Comparison of the evolutionary patterns of resistance in antibiotic- and AMP-resistant bacteria. (**E**) Fraction of polar amino acids and relative resistance level. (**F**) Fraction of positively charged and relative resistance levels. (**G**) AMP hydropathicity and relative resistance level. Each point shows the average MIC-fold change in the evolved MG1655 lines exposed to one of eight AMPs. (**H**) Single nucleotide polymorphisms (SNPs) of eight antibiotic-resistant strains compared to the original MG1655. (**I**) SNPs of ten AMP-resistant strains compared to the original MG1655.

To investigate the patterns of resistance evolution for antibiotics and AMPs, we selected a diverse panel of eight common antibiotics, spanning β-lactam, macrolides, amphenicols, fluoroquinolones, tetracyclines, aminoglycosides, nitrofurans, and sulfonamide synergists (Table S1), along with eight natural AMPs in clinical trial stages and two artificial AMPs synthesized in our laboratory (Table S2). Employing *E. coli* K-12 MG1655 (abbreviated as MG1655) as the model strain, we conducted experimental evolution in Mueller–Hinton (MH) broth with sub-inhibitory concentrations of these drugs over 60 days (Fig. 1A). The changes in minimum inhibitory concentration (MIC) values of MG1655 exposed to different drugs are presented in Tables S3 and S4. We found that MG1655 evolved significant resistance to all antibiotics, particularly to ciprofloxacin and kanamycin, with a 256-fold increase in MIC values (Fig. 1B). In contrast, resistance to most AMPs was not observed in MG1655, except for a marginal increase in resistance to colistin, SAAP-148, and SLAP-S25 (Fig. 1C). The results of the comprehensive comparative analysis showed that the rate and degree of bacterial resistance to antibiotics were significantly stronger than those to AMPs (*P* < 0.05) (Fig. 1D), suggesting a greater propensity for MG1655 to develop resistance to antibiotics.

To investigate the reasons accounting for the differential resistance evolution of AMPs, we performed a correlation analysis between the intrinsic physicochemical properties of AMPs and the rate of resistance evolution. By integrating the ratios of charged, polar, and hydrophobic amino acids in each AMP with the resistance phenotype observed during experimental evolution, we identified that AMPs with fewer polar (Fig. 1E) and positively charged amino acids (Fig. 1F), along with higher hydrophilicity (Fig. 1G), were less susceptible to resistance development.

To ensure the stable resistance evolution and inheritance of MG1655 under different drugs, we randomly selected three monoclonals from the evolved strains. The final antibiotic-evolved strains were designated as AMP120, AZI120, CHL80, CIP120, DOX120, KAN80, NIT70, and TMP30, while the final AMP-evolved strains were named COL120, Ind120, Omi120, Pex120, PGLA120, Pleu120, R8120, SAAP120, LI120, and SLAP120. The MIC values across different monoclonals were highly consistent (Fig. S1), indicating that the drug-exposed strains of MG1655 were both stable and inheritable.

To further dissect the mechanisms underlying resistance to antibiotics and AMPs, we conducted whole-genome sequencing (WGS) on the resistant strains. All evolved strains exhibited SNPs compared to the original MG1655 (Fig. 1H and I). Interestingly, many SNPs were corresponded with previously reported common resistance mechanisms in *E. coli* (Table S5). Among these SNPs, outer membrane porin OmpF, multidrug efflux pump AcrAB–TolC, and multidrug resistance regulators EmrR and MarR can mediate multidrug resistance in *E. coli* (25–27). The mutation of DNA gyrase-encoding gene *gyrA* mediates quinolone resistance in *E. coli* (28), and the oxygen-insensitive NADPH nitroreductase confers resistance to nitrofuran antibiotics (29). Notably, several final AMP-evolved strains (Ind120, Omi120, PGLA120, and Pleu120) exhibited SNPs despite no change in their MIC values (Table S6).

### Comparison of the fitness costs of antibiotic- and AMP-evolved strains

**Fig 2:**
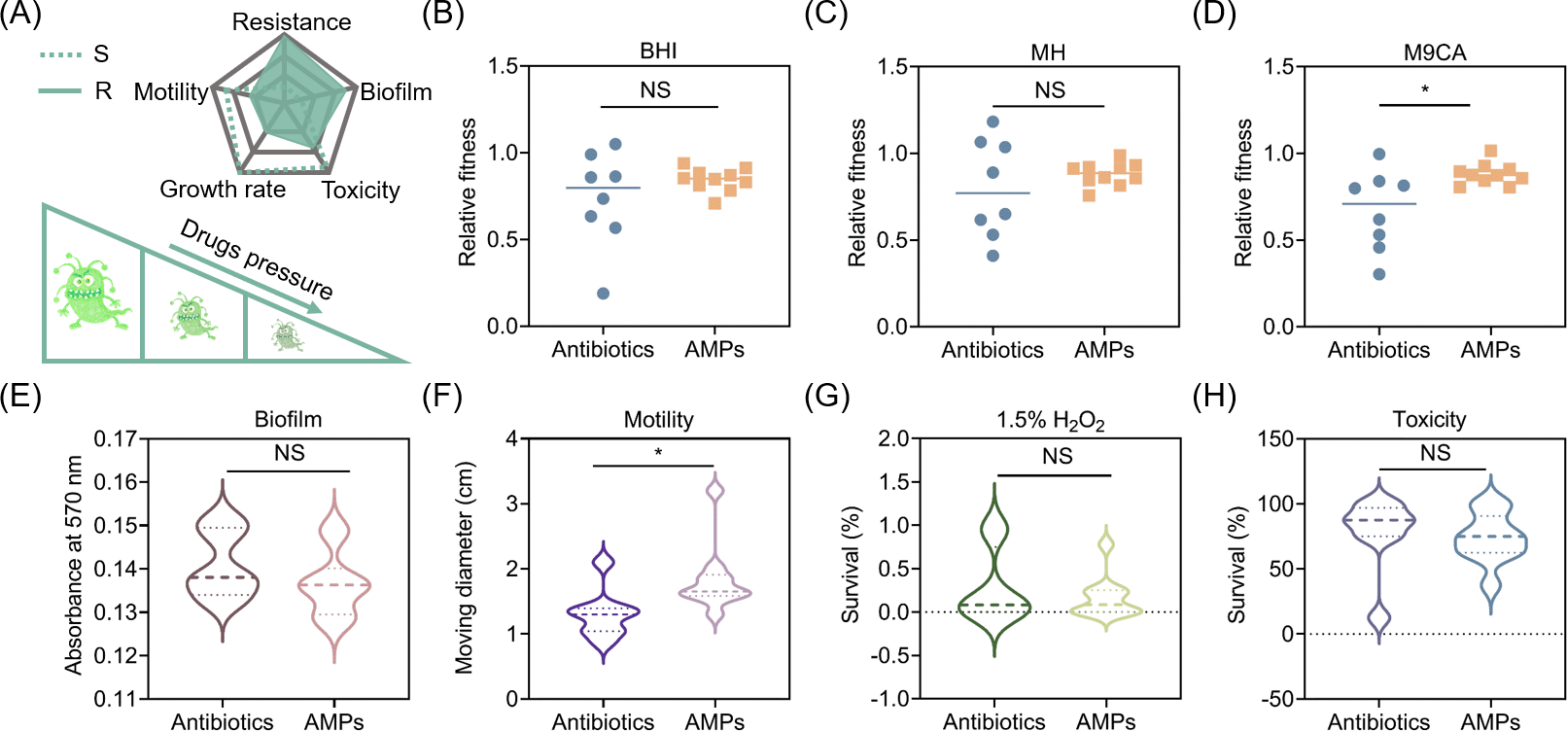
Fitness cost analysis of antibiotic- and AMP-resistant strains. (**A**) Schematic diagram of fitness cost patterns. The dotted line represents sensitive strains, and the solid line represents drug-resistant strains. (**B–D**) Comparison of relative fitness of antibiotic- and AMP-evolved strains in BHI broth (**B**), MH broth (**C**), and M9CA broth (**D**). The ratio of the area under the growth curve of the antibiotic- and AMP-evolved strains to the area under the growth curve of MG1655 determined the changes in relative fitness. (**E–H**) Comparison of the difference in biofilm-forming ability (**E**), swimming motility (**F**), antioxidant capacity (**G**), and *in vivo* toxicity (**H**) between antibiotic- and AMP-evolved strains. Lines represent the mean values. Statistical analyses were conducted with unpaired, two-tailed *t*-test (*P < 0.05). NS, not significant.

Bacteria can evolve resistance to antibiotics via genetic mutations or horizontal gene transfer, but this is often accompanied by fitness costs (Fig. 2A). To assess the effect of resistance evolution on bacterial growth, we monitored the growth curves of evolved strains across various nutrient media, with MG1655 serving as a control. The medium involved BHI broth (high-nutrient medium), MH broth (medium-nutrient broth), and M9CA broth (low-nutrient medium) that provide only carbon sources. Consistently, these evolved strains displayed superior growth in BHI broth and inferior growth in M9CA broth (Fig. S2A through F). Furthermore, we assessed the relative fitness of antibiotic- and AMP-evolved strains by calculating the ratio of the area under the growth curve of the final evolved strains to that of MG1655. In the three nutrient media, the relative fitness of most antibiotic- and AMP-evolved strains was lower than that of the original MG1655, suggesting a fitness cost associated with resistance evolution. Notably, while there was no significant difference in the relative fitness between antibiotic- and AMP-evolved strains in BHI and MH broth (Fig. 2B and C), the relative fitness of AMP-evolved strains in M9CA broth was significantly higher than that of antibiotic-evolved strains (Fig. 2D).

Biofilms, which are structured communities of bacterial cells enclosed in a self-produced polymer matrix, enable bacteria to survive harsh environments and are linked to chronic or persistent infections. Biofilms also protect bacteria from the host’s immune response and reduce sensitivity to antimicrobials and disinfectants (30). We compared the biofilm formation ability of MG1655 and its evolved strains using crystal violet staining. The results indicated that continuous sub-inhibitory concentrations of drugs did not affect the biofilm-forming ability of MG1655 (Fig. S3A and B), and there was no significant difference in biofilm-forming ability between antibiotic- and AMP-evolved strains (Fig. 2E), suggesting that biofilm formation is not associated with the evolution of drug resistance.

Bacterial motility, which aids in escaping antibiotic persecution, is closely related to the invasiveness and adhesion of pathogenic bacteria (31). Compared with MG1655, the motility of all antibiotic-evolved strains decreased significantly, except for AMP120 (Fig. S3C). In contrast, most AMP-evolved strains showed no significant change, while SLAP120 exhibited reduced motility, and Pex120 exhibited increased motility (Fig. S3D). Overall, the motility of antibiotic-evolved strains was significantly lower than that of AMP-evolved strains (Fig. 2F).

The production of reactive oxygen species (ROS) plays a crucial role in antibiotic-mediated killing of bacteria (32). In response to oxidative stress, bacteria have evolved self-antioxidant mechanisms. We compared the ability of MG1655 and the evolved strains to withstand hydrogen peroxide (H_2_O_2_) treatment. The results showed that 1.5% H_2_O_2_ had a strong killing effect on both antibiotic- and AMP-evolved strains, and the survival rate of all strains was lower than 1% (Fig. S3E and F). However, there was no significant difference in the ability of antibiotic- and AMP-evolved strains to resist H_2_O_2_ (Fig. 2G).

Furthermore, we used a *Galleria mellonella* larvae model to compare the virulence of MG1655 and the evolved strains of antibiotics and AMPs (Fig. 4A). Compared with the original MG1655, CIP120 and DOX120 strains resulted in decreased lethality, while AMP120, KAN80, and NIT70 showed increased lethality. Interestingly, NIT70 reduced the survival rate of *G. mellonella* by 80% (Fig. S3G). Continuous drug stimulation rendered most of the AMP-evolved strains, including COL120, Ind120, Pex120, PGLA120, Pleu120, R8120, and SAAP120, more virulent than the original MG1655 (Fig. S3H). Nevertheless, there was no significant difference in virulence between antibiotic- and AMP-evolved strains (Fig. 2H). Together, our results demonstrate that antibiotic-evolved strains are slightly worse off than AMP-evolved strains in restricted media and motility.

### Collateral sensitivity of antibiotic-resistant strains to diverse antibiotics and AMPs

**Fig 3:**
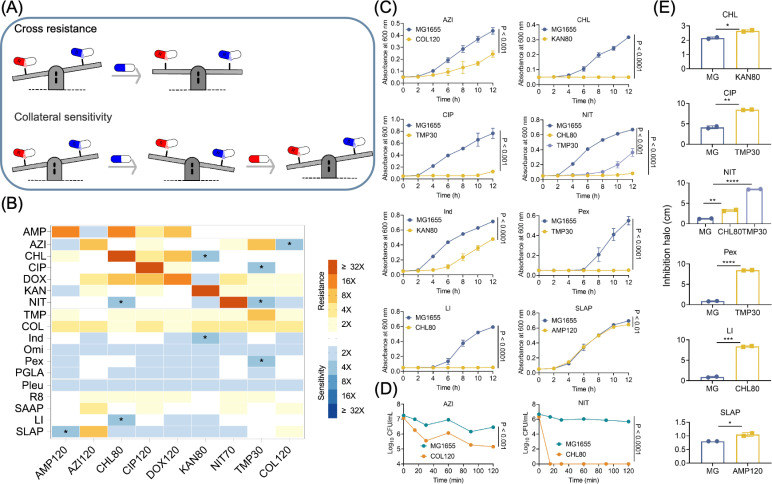
Susceptibility evaluation of antibiotic-resistant strains. (**A**) Schematic diagram of cross-resistance and collateral sensitivity patterns. (**B**) Heatmap of cross-resistance and collateral sensitivity of antibiotic-resistant strains to different antibiotics and AMPs. Cross-resistance is indicated in red, and collateral sensitivity is indicated in blue. Drugs that produced collateral sensitivity, for which the fold change of MIC was greater than or equal to four times, are indicated by asterisks (*). (**C**) Bacterial growth of a wild-type strain and antibiotic-resistant strains at a bacteriostatic concentration. MIC values of drugs to antibiotic-resistant strains were selected as the tested concentrations. (**D**) Time-dependent killing curves of MG1655 and antibiotic-resistant strains at lethal drug concentration. Tenfold MIC of drugs to MG1655 were selected as the drug concentration. (**E**) Comparison of the diameter of inhibition halo for MG1655 and antibiotic-resistant strains exposed to a drug concentration of 2,048 µg/mL. Data are shown as mean ± SD from three biological replicates. Statistical analyses were conducted with unpaired, two-tailed *t*-test (*P < 0.05, **P < 0.01, ***P < 0.001, ****P < 0.0001). NS, not significant.

Since the speed of new drug development lags far behind the evolution of bacterial drug resistance, collateral sensitivity-based therapy provides a promising solution to combat antibiotic resistance (Fig. 3A). Therefore, we assessed the susceptibility profiles of eight antibiotic-resistant strains and colistin-resistant strains against a panel of other antimicrobial agents or AMPs by comparing their MIC values with those of the wild-type MG1655 strain (Fig. 3B; Tables S7 and S8). The results revealed that cross-resistance predominantly occurred among antibiotics, while most antibiotic-resistant bacteria exhibited significant collateral sensitivity to multiple AMPs (Fig. 3B). Subsequently, we selected four antibiotic–antibiotic and five antibiotic–AMP pairs demonstrating collateral sensitivity exceeding fourfold for further investigation, including CHL80–NIT, KAN80–CHL, TMP30–NIT, TMP30–CIP, COL120–AZI, AMP120–SLAP, CHL80–LI, KAN80–Ind, and TMP30–Pex (Fig. 3B).

To verify the collateral sensitivity of these nine pairs, we determined bacterial growth curves of MG1655 and antibiotic-resistant bacteria exposed to corresponding collateral sensitivity drugs. Consistently, the growth of antibiotic-resistant strains was more readily inhibited than that of the original MG1655 at the same concentration (Fig. 3C). Notably, the growth of KAN80, TMP30, and CHL80 was completely inhibited, whereas the original MG1655 strain showed normal growth. Furthermore, we conducted killing kinetics of two antibiotic-resistant strains under lethal concentrations of collateral-sensitive drugs at 10-fold MIC. Upon lethal drug concentration stress, collateral-sensitive populations showed a significantly more rapid CFU reduction compared with MG1655. In particular, a 15-min nitrofurantoin exposure completely eradicated chloramphenicol-resistant bacteria, while the numbers of original MG1655 were decreased by less than one-log at all (Fig. 3D). Concurrently, disk diffusion experiments confirmed these results, revealing that the inhibition halo diameters in antibiotic-resistant strains were significantly increased compared with those of MG1655 (Fig. 3E). Among them, nitrofurantoin and pexiganan had a significant inhibitory effect on trimethoprim-resistant strains, and LI14 displayed a noticeable inhibitory effect on chloramphenicol-resistant strains.

**Fig 4:**
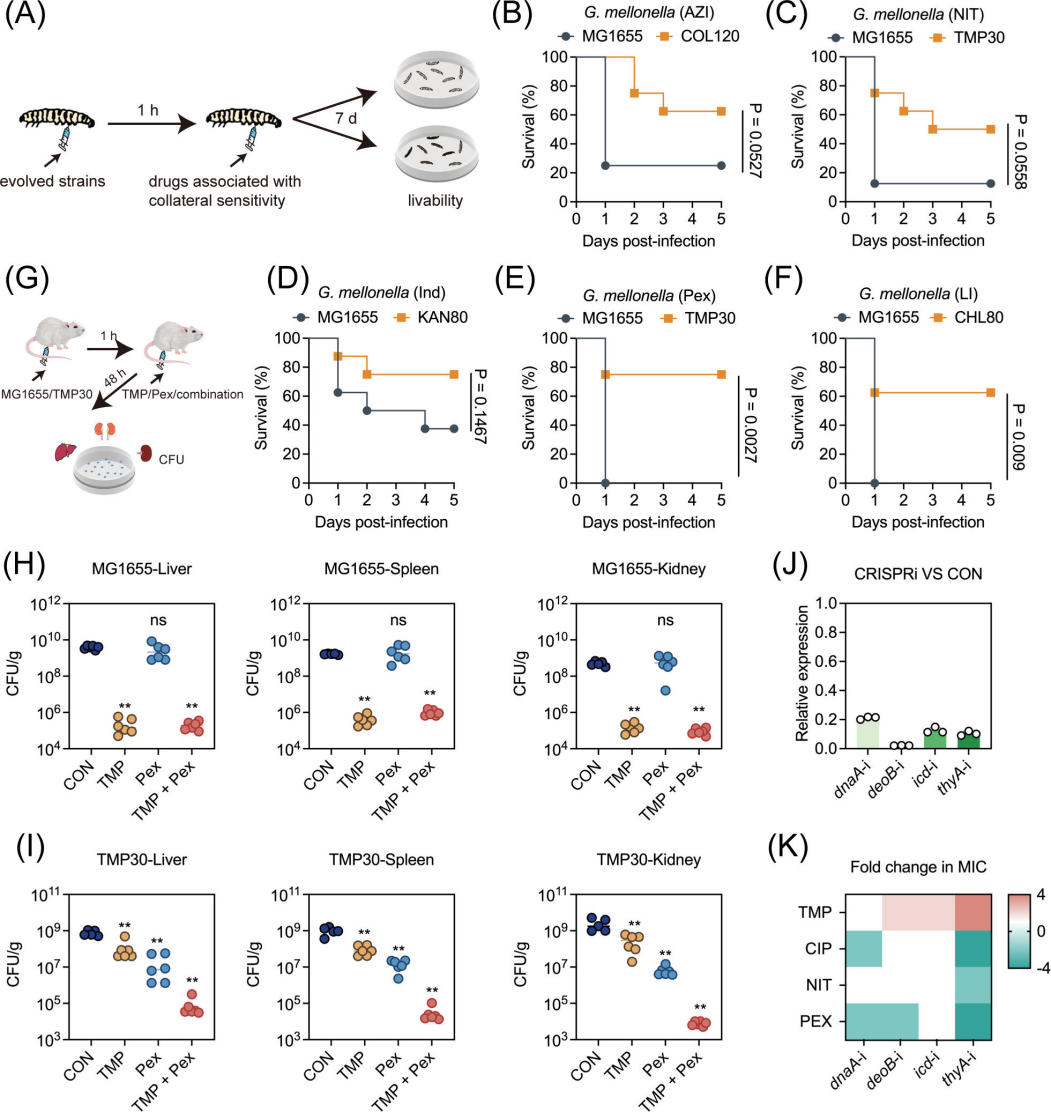
Collateral sensitivity combinations display strong anti-infective potential. (**A**) Schematic diagram of *G. mellonella* infection patterns. Survival of *G. mellonella* larvae (*n* = 8 per group) infected with MG1655 and antibiotic-resistant strains and treated with azithromycin (**B**), nitrofurantoin (**C**), indolicidin (**D**), pexiganan (**E**), LI14 (**F**) at 30 mg/kg 5 days post-infection. *P* values were determined by log-rank (Mantel–Cox) test. (**G**) Schematic diagram of mouse peritonitis infection patterns. (**H**) Bacterial loads in the liver, spleen, and kidneys of mice infected with MG1655 and treated with PBS, TMP, Pex, or a combination of both. (**I**) Bacterial loads in the liver, spleen, and kidneys of mice infected with TMP30 and treated with PBS, TMP, Pex, or a combination of both. Statistical significance was assessed by the nonparametric one-way analysis of variance and denoted as follows: *P < 0.05; **P < 0.01; ***P < 0.001; ns, not significant. (**J**) Relative expression of *dnaA*, *deoB*, *icd*, and *thyA* genes in MG1655 after CRISPR interference. (**K**) Heatmap of the fold change in MIC of trimethoprim, ciprofloxacin, nitrofurantoin, and pexiganan to gene repression-MG1655 after CRISPR interference compared with the original MG1655.

Next, we established a *G. mellonella* infection model to assess the *in vivo* efficacy of collateral sensitivity-pair drug combinations (Fig. 4A). As expected, the survival rates of larvae infected with antibiotic-resistant strains were significantly improved after treatment with the corresponding collateral sensitivity drugs. Specifically, pexiganan or LI14 monotreatment achieved 75% or 62.5% of the survival rate against trimethoprim- or chloramphenicol-resistant strains, respectively, whereas all larvae infected by the original MG1655 died (Fig. 4B through F). To further evaluate the feasibility of therapeutic strategies based on collateral sensitivity, TMP30 was selected for further validation in a mouse peritonitis infection model (Fig. 4G). Consistent with the results of the *G. mellonella* model, the bacterial loads in the liver, spleen, and kidneys of mice infected with MG1655 decreased significantly with trimethoprim monotherapy or in combination with pexiganan, contrasting with pexiganan monotherapy (Fig. 4H). Conversely, mice infected with TMP30 and treated with pexiganan monotherapy or in combination with trimethoprim had significantly lower bacterial loads than trimethoprim monotherapy (Fig. 4I). To explore the mechanisms underlying the collateral sensitivity of TMP30 to pexiganan, we screened four candidate genes from the SNP results, including *dnaA*, *deoB*, *icd*, and *thyA*, and constructed their gene expression-repression strains via clustered regularly interspaced short palindromic repeats interference (CRISPRi) (Fig. 4J). Consequently, we found that only *thyA-*i strains showed increased resistance to trimethoprim (four-fold increase in MIC), while simultaneously displaying increased sensitivity to trimethoprim, ciprofloxacin, nitrofurantoin, and pexiganan (four-, two-, and four-fold decreases in MIC) (Fig. 4K; Tables S9 and S10), implying that mutations in *thyA* encoding thymidylate synthase (33) may be responsible for the collateral sensitivity of TMP-resistant strains to pexiganan.

## DISCUSSION

Under the pressure of global antibiotic restriction, AMPs with diverse physiochemical features and lower resistance have become favored in clinical medicine (11). However, the limited bioavailability and rapid degradation of AMPs in physiological environments hinder their clinical application. Through appropriate chemical modification and optimization, the stability of AMPs can be improved, thereby broadening their scope and efficiency in practical applications. For example, nanocarriers have been explored to alter the delivery of AMPs by responding to dynamic changes in microenvironmental factors, including pH and ROS (34).

The slower evolution of AMP resistance compared with antibiotics, along with their potential immunomodulatory abilities, underscores the value of AMPs in the era of increasing drug resistance (35, 36). Moreover, we recently highlight the potential of AMPs as promising antibiotic adjuvants to combat drug-resistant pathogens (37), offering novel combinational options for combating antibiotic-resistant bacterial infections.

In our study, we tracked the evolutionary trajectory of bacterial resistance to antibiotics and AMPs using the model strain *E. coli* MG1655. Our study corroborated that bacteria develop resistance to antibiotics significantly faster than to AMPs, aligning with previous findings (38). The antibacterial activity of most AMPs used in this study is highly dependent on electrostatic interactions with bacterial membrane, which may explain the rarity of AMP resistance. There were notable differences in the rate of resistance evolution among various antibiotics, with MG1655 showing the slowest resistance development to azithromycin. This could be attributed to azithromycin’s unique interaction with the outer membrane of Gram-negative bacteria and its distinct binding dynamics with the 50S ribosome subunit (39). Despite long-term exposure, widespread resistance to AMPs remains uncommon. However, the emergence of bacterial resistance is an inevitable outcome of pathogen evolution. For example, the plasmid-mediated colistin resistance gene *mcr-1*, reported in 2016, mediates resistance to polymyxins, a class of cationic lipopeptide antibiotics (40). Our study further deciphered antimicrobial resistance patterns in conjunction with the inherent physicochemical properties of AMPs, revealing that AMPs with higher polarity, positive charge, and lower hydrophilicity were more prone to resistance development. These findings inspire the design of novel AMPs that are less susceptible to resistance.

Generally, the evolution of drug resistance imposes a significant burden on host bacterial strains, reducing the competitiveness of resistant bacteria in the absence of antibiotic pressure (14–16). In our study, the acquisition of resistance indeed delayed or reduced the growth and pathogenicity of most bacteria. Most notably, nitrofurantoin-resistant strains displayed high levels of resistance and enhanced virulence, suggesting a potential risk associated with nitrofurantoin resistance. Interestingly, despite 120 subcultures, we did not induce significant resistance to most AMPs. However, the AMP-evolved strains still showed fitness cost-related changes, particularly in bacterial growth.

The epidemic spread of bacterial resistance has seriously limited the effectiveness of clinical antibiotic therapy, highlighting the urgent need for novel antimicrobial strategies. Collateral sensitivity, a phenomenon where resistance to certain classes of antibiotics increases sensitivity to others (41), suggests that rational antibiotic rotation can effectively treat infections caused by resistant strains (23). However, collateral sensitivity is not conserved across different genetic backgrounds, and its randomness significantly limits its potential application in clinical settings. Understanding the key molecular mechanisms underlying collateral sensitivity is crucial not only for elucidating its role in antimicrobial therapy but also for guiding the development of multi-directional drug strategies (18). In this study, we comprehensively explored the collateral sensitivity of antibiotic-resistant *E. coli* to various antibiotics and AMPs. Our findings revealed that while antibiotic-resistant *E. coli* showed increased resistance to most antibiotics, they displayed collateral sensitivity to multiple AMPs, which is consistent with a previous study (42). Specifically, we demonstrated that ampicillin-resistant *E. coli* strains showed enhanced susceptibility to the short-linear AMP SLAP-S25, and chloramphenicol-resistant *E. coli* strains exhibited increased sensitivity to LI14, which was discovered in our laboratory (43). It has been concluded that the frequency of cross-resistance is usually two to three times higher than that of collateral sensitivity (42, 44, 45). Intriguingly, we found that the collateral sensitivity of antibiotic-resistant bacteria to AMPs was nearly three times higher than that of cross-resistance, suggesting that AMPs may represent a particularly effective class of antimicrobial agents for leveraging collateral sensitivity. In our investigation of the collateral sensitivity of antibiotic-resistant bacteria to antibiotics, we found that nitrofurantoin had the highest frequency of collateral sensitivity among the selected antibiotics. In agreement with our results, a previous study also found that drug-resistant mutants of tigecycline, methicillin, and protamine all showed collateral sensitivity to nitrofurantoin (46). The widespread collateral sensitivity to nitrofurantoin may be related to its ability to induce DNA damage and trigger the typical SOS response in bacteria (47–49).

In addition, it has been shown that mutations in the TrkH gene, which encodes the potassium ion transporter protein, can diminish the functionality of the AcrAB–TolC efflux pump by reducing proton motive force (21). This resulted in unidirectional collateral sensitivity of aminoglycoside-resistant *E. coli* to doxycycline, lomefloxacin, ciprofloxacin, trimethoprim, and sulfamethoxil (22). The activity of the TrkH ion channel can be modulated by ATP-induced conformational changes in the TrkA protein (50), and consistently, a mutation of Trk potassium uptake system protein TrkA in kanamycin-evolved strains was observed. Regarding the widespread collateral sensitivity of antibiotic-resistant bacteria to AMPs, we speculated that this phenomenon may be partly due to mutations in typical resistance genes that frequently emerge under various antibiotic stresses. One notable example is the *mar*-regulated transcription suppressor (*marR*), which undergoes non-synonymous mutations in chloramphenicol-resistant strains. Consistently, mutations in *marR* have been shown to increase the surface negative charge of the bacterial outer membrane, ultimately leading to increased sensitivity to several cationic AMPs (42). Although we extensively compared the collateral sensitivity of antibiotic-resistant bacteria to AMPs, there remain limitations in extending these findings beyond *E. coli* or controlled laboratory settings. Moreover, more studies are warranted to decipher the specific molecular mechanisms underlying the collateral sensitivity between antibiotics and AMPs.

In conclusion, our study tracks the evolutionary trajectory of bacterial resistance to both antibiotics and AMPs, revealing that AMPs have a lower propensity to develop resistance. The acquisition of resistance often imposes fitness costs on the bacterial strains, yet certain drug-resistant strains display enhanced virulence. Notably, our work identifies nine collateral sensitivity combinations, especially between antibiotics and AMPs, and confirms their efficacy. These findings offer a promising strategy to combat the escalating crisis of antibiotic resistance.

## MATERIALS AND METHODS

### Synthesis and validation of peptides

All peptides described herein were synthesized by GL Biochem Company (Shanghai, China) using solid-phase polypeptide synthesis, with their precise molecular weight determined through matrix-assisted laser desorption/ionization time-of-flight mass spectrometry. The obtained peptides exhibited a purity exceeding 95%.

### Minimum inhibitory concentration (MIC) determination

The broth microdilution method was used to measure the MIC values of antibiotics and peptides according to the Clinical and Laboratory Standards Institute guidelines. The MIC value is defined as the lowest drug concentration without visible bacteria.

### Experimental evolution experiments

Eight antibiotics and ten AMPs at the sub-inhibitory concentration were prepared. A 300-µL system, including 260 µL of MH broth, 30 µL of prepared drug, and 10 µL of bacterial culture, was used for resistance passages. After 12 h of incubation at 37℃ and 200 rpm, 10 µL of the above bacterial culture was added into the new sterile drug-containing broth. MIC values of bacterial culture were determined. The drug concentration was adjusted according to the MIC value change until the end of the 120 passages. The passages were stopped in advance when the MIC values reached 512 µg/mL.

### Genome extraction and whole-genome sequencing of evolved strains

The FastPure Bacteria DNA Isolation Mini Kit was used to extract the total DNA of MG1655 and the final evolved strains. The extracted genome was sent to GENEWIZ (Suzhou, China) for Illumina high-throughput sequencing. After obtaining the sequencing data, SPAdes was used to separately assemble the short-read Illumina raw sequences of original and evolved strains, and contigs less than 500 bp were discarded (51). Snippy (4.0.2) was used to perform SNP analysis against the genome of the original strain (52).

### Biofilm formation assay

Logarithmic phase cells were diluted 1,000-fold in MH broth, 200 µL of mixture was added into a 96-well flat plate and incubated at 37°C for 48 h. Then, the bacterial suspension in the 96-well flat plate was gently discarded. Plankton bacteria on the surface of the biofilm were removed using sterile saline three times. Biofilm was fixed with 50 µL of methanol for 15 min and stained with 100 µL of 0.1% crystal violet for another 15 min. After three rinses with saline, 100 µL of freshly prepared 33% acetic acid was added. Subsequently, the absorbance of the mixture at a wavelength of 570 nm was measured using a microplate reader (Tecan).

### Swimming motility analysis

Bacteria were cultured at 37°C and 200 rpm to the logarithmic phase (1 × 10^6^ CFUs). Two microliters of bacterial solution was inoculated in the center of a 0.4% LB agar plate. After culturing at 37°C for 48 h, the diameter of the bacterial circle centered on the inoculation position was measured.

### Antioxidant capacity determination

Bacterial cells were proportionally mixed with 3% hydrogen peroxide solution and incubated at 37°C for 1 h in the dark. The reaction was terminated by adding a catalase solution with a final concentration of 1,000 U/mL. After diluting the mixture to an appropriate gradient, the drops were carried out on LB agar plates, and then the agar plates were placed in a 37°C incubator overnight for colony counting.

### *Galleria mellonella* infection model

The bacterial culture was uniformly diluted with saline to an OD_600_ of 0.35. *G. mellonella* larvae (*n* = 8 per group) were randomly divided, and sterile saline was used as the blank control. In the virulence assessment, 10 µL of bacterial solution was inoculated into each larva using a Hamilton syringe. The survival of larvae was observed and recorded daily for 7 days.

For the larval infection protection model, larvae were infected with wild-type and antibiotic-resistant strains and treated with a dose of 30 mg/kg of drugs at the left foot of the last gastropod at 1 h post-infection, and the survival was monitored for 5 days.

### Time-dependent killing assay

Bacteria were cultured at 37°C and 200 rpm to the logarithmic phase (1 × 10^6^ CFU). The corresponding drugs, with a final concentration of tenfold the MIC of MG1655, were added. At 15, 30, 60, 90, and 120 min, 50 µL of the mixture was serially diluted 10-fold and dropped on an LB agar plate for CFU counting.

### Disk diffusion experiment

Fifty microliters of bacterial cultures was spread evenly onto LB agar. A prepared circular sterile filter paper was pasted on the center of the petri dish, and the corresponding drugs with a concentration of 2,048 µg/mL were dropped on the center of the circular filter paper. The petri dishes were placed at 37°C for 24 h, and the diameters of the sterile growth circles were measured.

### CRISPR interference

The engineered CRISPR/CRISPR-associated protein 9 (Cas9) system was used to repress the *dnaA*, *deoB*, *icd*, and *thyA* genes. Briefly, spacer 1 (5-CAGGTGGCGGATAACCCTGG-3), spacer 2 (5-TGATGAAAAACACGGCCAGG-3), spacer 3 (5-GAAGGAGCGTTTAAAGACTG-3), and spacer 4 (5-GGCCAGTGTATGGTAAACAG-3) were synthesized, and then, the construction of the targeting plasmid psgRNA was performed in a total volume of 25 µL. Subsequently, 1.5 µL of each newly constructed psgRNA and dCas9 was simultaneously electroporated into the competent cells of MG1655. MG1655 containing unmodified psgRNA and dCas9 was used as a control. Anhydrotetracycline at a final concentration of 0.5 µg/mL was used to induce gene repression. RT-qPCR was used to verify the expression of the genes.

### RT-qPCR analysis

The 7500 Fast Real-time PCR System (Applied Biosystems, California, USA) was used to perform RT-qPCR analysis using the ChamQ SYBR color qPCR master mixing kit (Vazyme Biotech, China) with the optimized primers (Table S10). The internal reference gene (16S rRNA) was used to perform relative quantitative calculation of multiple changes in mRNA expression. The mRNA expression levels of the genes were calculated using the −∆ΔCt method.

### Mouse peritonitis infection model

Female BALB/c mice aged 6 to 8 weeks were randomly divided into four groups with six replicates for each group. Of bacterial suspension, 5.0 × 10^8^ CFU was intraperitoneally injected to mice for 1 h, followed by a second intraperitoneal injection of trimethoprim (60 mg/kg) or pexiganan (10 mg/kg) or a combination of trimethoprim and pexiganan (30 + 5 mg/kg). The PBS injection group was used as a control. After 48 h, the liver, spleen, and kidneys of the mice were aseptically removed for CFU measurement.

### Statistical analysis

Statistical analysis was performed using GraphPad version 9.0 software. All data were presented as mean ± SD, and *P* value was determined using unpaired *t*-test between two groups (*P < 0.05, **P < 0.01, ***P < 0.001, ****P < 0.0001; NS, not significant).

## SUPPLEMENTAL MATERIAL

Supplemental material msystems.01700-24-s0001.docx Supplemental figures and tables.

ASM does not own the copyrights to Supplemental Material that may be linked to, or accessed through, an article. The authors have granted ASM a non-exclusive, world-wide license to publish the Supplemental Material files. Please contact the corresponding author directly for reuse.
