# Supplementary material for: Evolutionary trajectory of bacterial resistance to antibiotics and antimicrobial peptides in Escherichia coli
Source: mSystems. 2025 Feb 27;10(3):e01700-24. doi: 10.1128/msystems.01700-24 (PMC11915801; doi:10.1128/msystems.01700-24)
Supplement: Supplemental material — Supplemental figures and tables. [file msystems.01700-24-s0001.docx]

**Supplementary Materials**

**Figures**


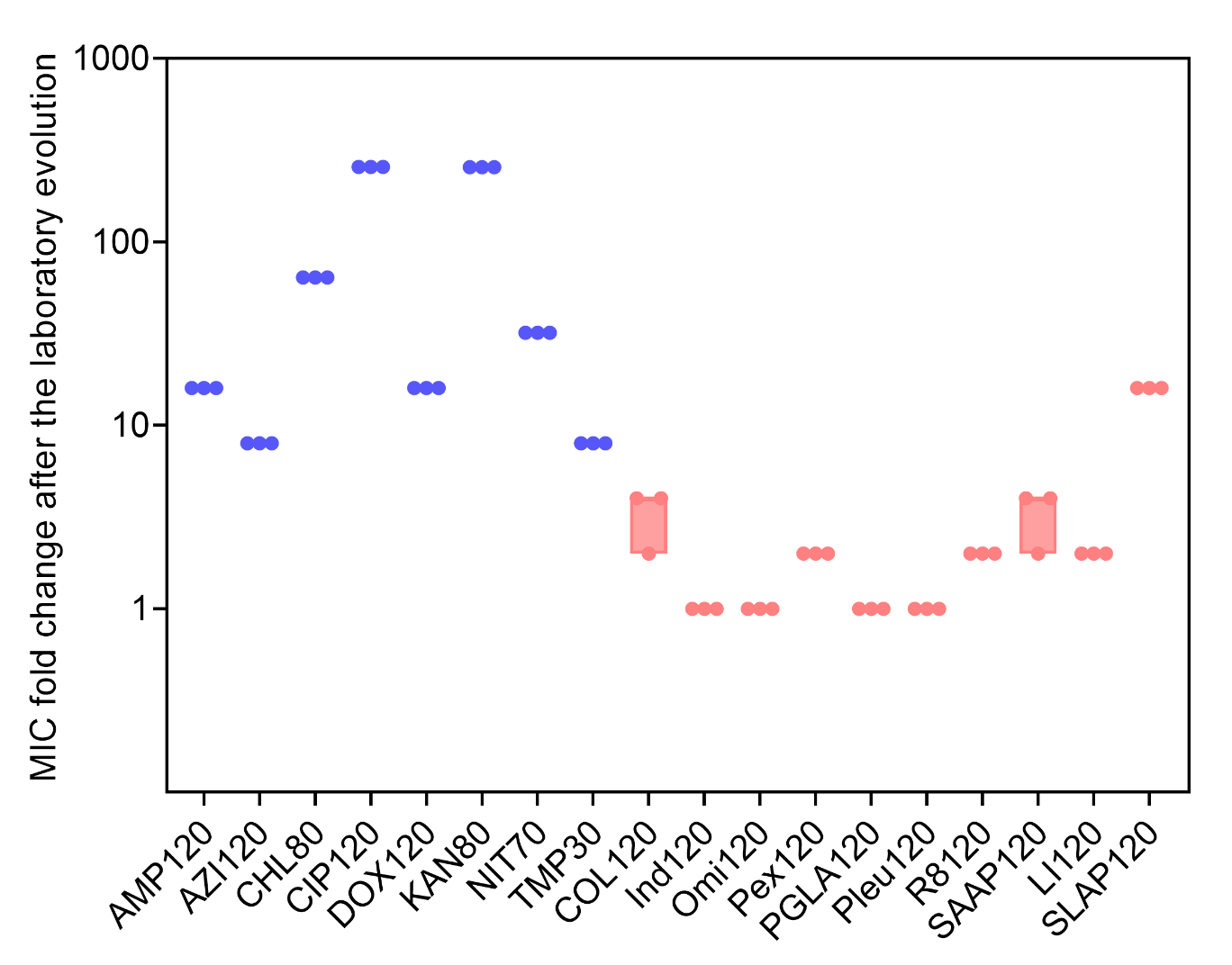


**Figure S1. The evolved MG1655 under drugs exposure are stable and inheritability.**

Resistance levels of monoclonals to different drugs after experimental evolution. The antibiotic-resistant strains were indicated in the left eight blue points and the AMP-resistant strains were indicated in the right ten red points.

**
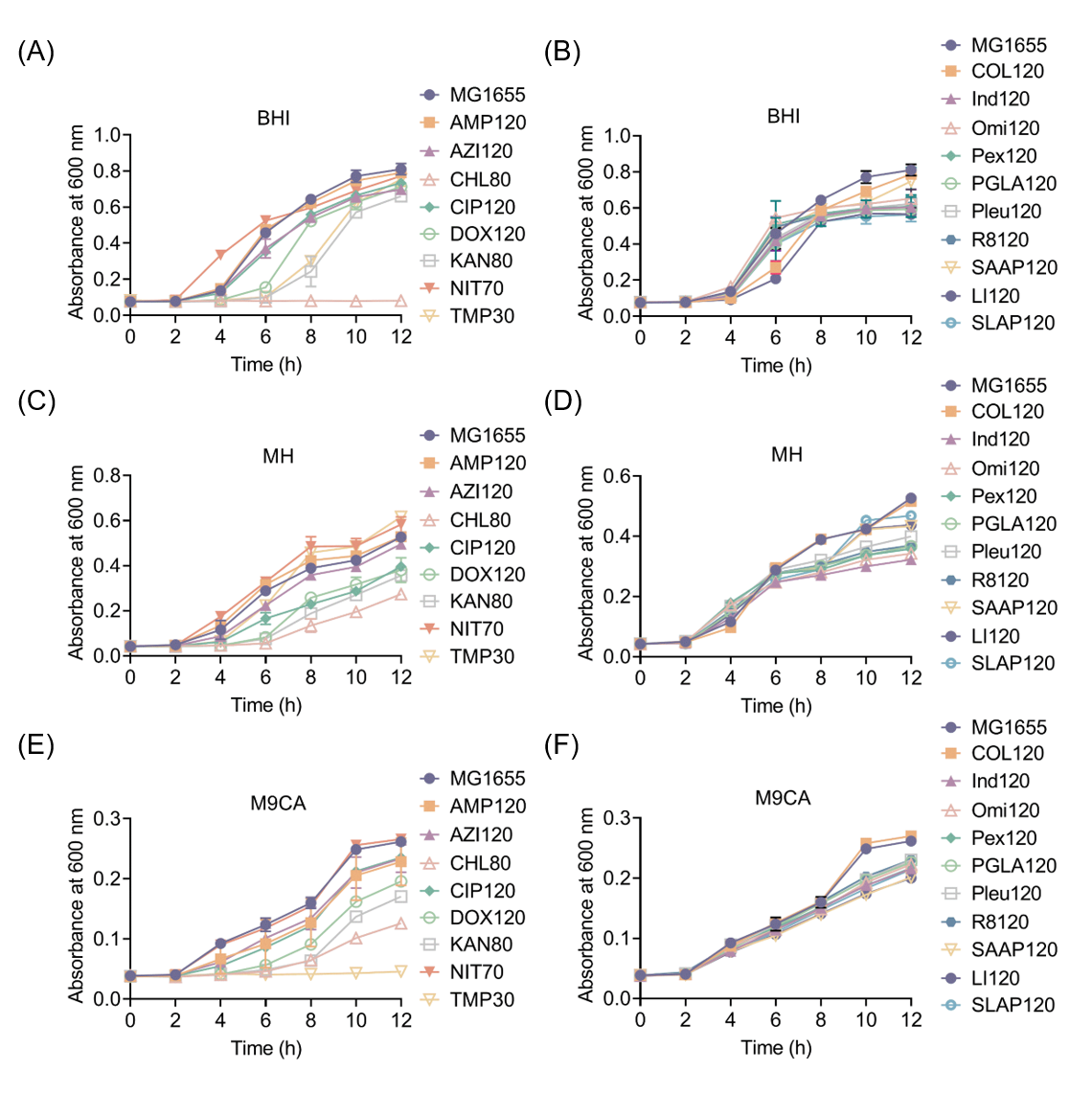
**

**Figure S2. Comparison of relative fitness of antibiotics- and AMPs-evolved strains in different nutrient medium.**

The growth curves of the final evolved strains of antibiotics and AMPs were measured in BHI broth **(A-B)**, MH broth **(D-E)**, M9CA broth **(G-H)** with MG1655 as a control. The ratio of the area under the growth curve of the antibiotics and AMPs final evolved strains to the area under the growth curve of MG1655 determined the changes of relative fitness.

**
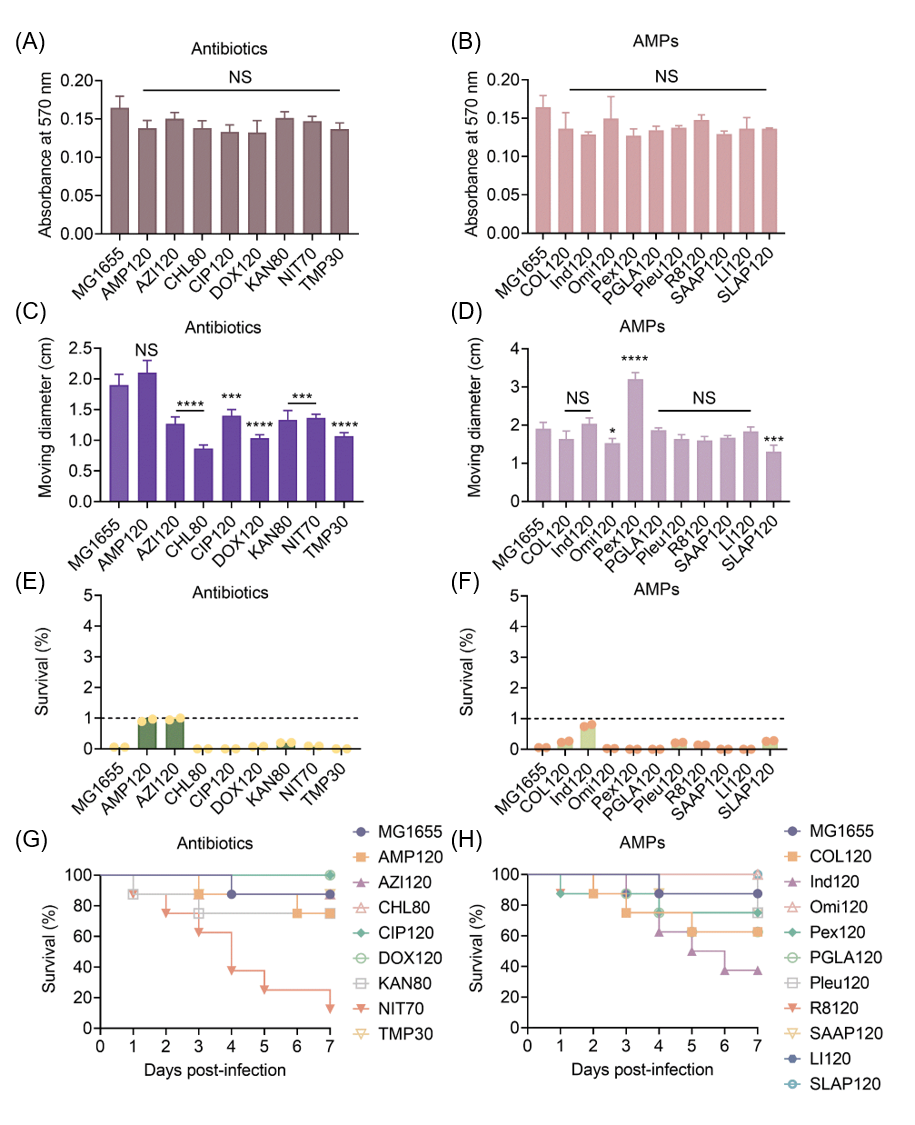
**

**Figure S3. Evaluation of fitness costs for antibiotics- and AMPs-evolved strains.**

**(A and B)** Biofilm formation ability of antibiotics- and AMPs-evolved strains. **(C and D)** The motility of antibiotics- and AMPs-evolved strains. **(E and F)** The antioxidant capacity of antibiotics- and AMPs-evolved strains. **(G and H)** Survival of *G. mellonella* larvae infected with MG1655 and antibiotics- and AMPs-evolved strains after 7 days post-infection. Data are shown as mean ± SD from three biological replicates. Statistical analyses were conducted with unpaired, two-tailed t-test (**P*≤ 0.05, ***P*≤ 0.01, ****P* ≤ 0.001, *****P* ≤ 0.0001). NS, not significant.

**Tables**

**Table S1. Information of antibiotics used in this study.**

| Abbrev. | Name | Mode of action | Type of action |
| --- | --- | --- | --- |
| AMP | Ampicillin | Cell wall | Bactericidal |
| AZI | Azithromycin | Protein synthesis, 50S | Bacteriostatic |
| CHL | Chloramphenicol | Protein synthesis, 50S | Bacteriostatic |
| CIP | Ciprofloxacin | Gyrase | Bactericidal |
| DOX | Doxycycline | Protein synthesis, 30S | Bacteriostatic |
| KAN | Kanamycin | Protein synthesis, 30S | Bactericidal |
| NIT | Nitrofurantoin | Multiple mechanisms | Bactericidal |
| TMP | Trimethoprim | Folic acid biosynthesis | Bacteriostatic |

**Table S2. Physicochemical properties of AMPs.**

| Abbrev. | Name | Source | Sequences (N to C) | Formula | MW | Net charge | H^a^ | μH^b^ | pl^c^ |
| --- | --- | --- | --- | --- | --- | --- | --- | --- | --- |
| COL | Colistin | Microorganism | Fatty acyl-Dab-Thr-Dab-Dab-Dab-_D_-Leu-Leu-Dab-Dab-Thr | C_55_H_96_N_16_O_13_ | 1189.45 | - | - | - | - |
| Ind | Indolicidin | Bovine | ILPWKWPWWPWRR | C_100_H_131_N_25_O_14_ | 1907.30 | +3 | 1.069 | 0.190 | 12.01 |
| Omi | Omiganan | Bovine | ILRWPWWPWRRK | C_90_H_126_N_26_O_13_ | 1780.16 | +4 | 0.827 | 0.290 | 12.30 |
| Pex | Pexiganan | Synthetic | GIGKFLKKAKKFGKAFVKILKK | C_122_H_209_N_31_O_23_ | 2478.20 | +9 | 0.241 | 0.674 | 10.90 |
| PGLA | Peptide Glycine Leucine Amide | Frog | GMASKAGAIAGKIAKVALKAL | C_88_H_161_N_25_O_23_S_1_ | 1969.46 | +4 | 0.363 | 0.353 | 10.48 |
| Pleu | Pleurocidin | Fish | GWGSFFKKAAHVGKHVGKAALTHYL | C_129_H_192_N_36_O_29_ | 2711.17 | +4 | 0.421 | 0.309 | 10.18 |
| R8 | R8 | Synthetic | FLGKVFKLASKVFKAVFGKV | C_111_H_177_N_25_O_22_ | 2213.78 | +5 | 0.553 | 0.570 | 10.60 |
| SAAP | SAAP-148 | Human | LKRVWKRVFKLLKRYWRQLKKPVR | C_155_H_258_N_48_O_27_ | 3226.06 | +11 | 0.301 | 0.823 | 12.19 |
| LI | LI14 | Synthetic | LKKLCRILKKLCRI | C_78_H_150_N_24_O_15_S_2_ | 1728.32 | +6 | 0.570 | 0.811 | 10.48 |
| SLAP | SLAP-S25 | Microorganism | AC-Dab-Ile-Dab-Ile-Dab-_D_-Phe-Leu-Dab-_D_-Val-Leu-Ala-NH_2_ | C_65_H_117_N_23_O_12_ | 1412.79 | +4 | 0.571 | 0.447 | 12.48 |

**Table S3. MIC changes of MG1655 under the pressure of antibiotics.**

| Generation | AMP | AZI | CHL | CIP | DOX | KAN | NIT | TMP |
| --- | --- | --- | --- | --- | --- | --- | --- | --- |
| primary | 4 | 2 | 8 | 0.0078 | 2 | 2 | 16 | 64 |
| 10 | 8 | 2 | 8 | 0.0156 | 16 | 16 | 16 | 128 |
| 20 | 8 | 2 | 16 | 0.0625 | 32 | 32 | 16 | 128 |
| 30 | 8 | 2 | 32 | 0.25 | 32 | 64 | 64 | 512 |
| 40 | 16 | 2 | 64 | 0.25 | 32 | 64 | 64 |  |
| 50 | 16 | 4 | 128 | 0.5 | 32 | 128 | 128 |  |
| 60 | 16 | 4 | 256 | 0.5 | 32 | 256 | 256 |  |
| 70 | 16 | 4 | 256 | 0.5 | 32 | 256 | 512 |  |
| 80 | 16 | 4 | 512 | 1 | 32 | 512 |  |  |
| 90 | 32 | 4 |  | 1 | 32 |  |  |  |
| 100 | 32 | 4 |  | 1 | 32 |  |  |  |
| 110 | 64 | 8 |  | 2 | 32 |  |  |  |
| 120 | 64 | 16 |  | 2 | 32 |  |  |  |

**Table S4. MIC changes of MG1655 under the pressure of AMPs.**

| Generation | COL | Ind | Omi | Pex | PGLa | Pleu | R8 | SAAP | LI | SLAP |
| --- | --- | --- | --- | --- | --- | --- | --- | --- | --- | --- |
| primary | 0.125 | 16 | 16 | 4 | 8 | 2 | 4 | 16 | 8 | 4 |
| 10 | 0.125 | 16 | 16 | 4 | 8 | 2 | 4 | 16 | 8 | 4 |
| 20 | 0.125 | 16 | 16 | 8 | 8 | 2 | 4 | 16 | 8 | 8 |
| 30 | 0.25 | 16 | 16 | 8 | 8 | 2 | 4 | 16 | 8 | 8 |
| 40 | 0.25 | 16 | 16 | 8 | 8 | 2 | 4 | 16 | 8 | 8 |
| 50 | 0.5 | 16 | 16 | 8 | 8 | 2 | 4 | 16 | 8 | 16 |
| 60 | 0.5 | 16 | 16 | 8 | 8 | 2 | 4 | 16 | 16 | 16 |
| 70 | 0.5 | 16 | 16 | 8 | 8 | 2 | 4 | 16 | 16 | 16 |
| 80 | 0.5 | 16 | 16 | 8 | 8 | 2 | 4 | 32 | 16 | 32 |
| 90 | 0.5 | 16 | 16 | 8 | 8 | 2 | 4 | 32 | 16 | 32 |
| 100 | 0.5 | 16 | 16 | 8 | 8 | 2 | 4 | 32 | 16 | 32 |
| 110 | 0.5 | 16 | 16 | 8 | 8 | 2 | 8 | 64 | 16 | 64 |
| 120 | 0.5 | 16 | 16 | 8 | 8 | 2 | 8 | 64 | 16 | 64 |

**Table S5. Single Nucleotide Polymorphism (SNPs) of antibiotics-evolved strains.**

| **Strains** | **SNPs** | |
| --- | --- | --- |
| AMP120 | IS1 protein InsA | |
|  | Cyclic AMP receptor protein | |
|  | Putative esterase YheT functionally coupled to phosphoribulokinase homolog | |
|  | Serine recombinase, PinQ/PinR-type | |
|  | Translation elongation factor Tu | |
|  | LSU ribosomal protein L22p (L17e) | |
|  | Lon protease homolog YcbZ | |
|  | H repeat-associated protein, YhhI family | |
| AZI120 | IS1 protein InsA | |
|  | Cyclic AMP receptor protein | |
|  | Putative esterase YheT functionally coupled to phosphoribulokinase homolog | |
|  | Serine recombinase, PinQ/PinR-type | |
|  | Translation elongation factor Tu | |
|  | LSU ribosomal protein L22p (L17e) | |
|  | Lon protease homolog YcbZ | |
|  | H repeat-associated protein, YhhI family | |
| CHL80 | IS1 protein InsA | |
|  | Cyclic AMP receptor protein | |
|  | Putative esterase YheT functionally coupled to phosphoribulokinase homolog | |
|  | Aerobic respiration control sensor protein ArcB | |
|  | Multiple antibiotic resistance protein MarR | |
|  | Serine recombinase, PinQ/PinR-type | |
|  | Multidrug efflux system AcrAB-TolC, inner-membrane proton/drug antiporter AcrB | |
|  | Translation elongation factor Tu | |
|  | Phage head, terminase subunit Nu1 | |
| CIP120 | | IS1 protein InsA |
|  |  | Cyclic AMP receptor protein |
|  |  | Putative esterase YheT functionally coupled to phosphoribulokinase homolog |
|  |  | Aerobic respiration control response regulator ArcA |
|  |  | Multiple antibiotic resistance protein MarR |
|  |  | Uncharacterized protein, YnaE family |
|  |  | Serine recombinase, PinQ/PinR-type |
|  |  | DNA gyrase subunit A |
|  |  | DNA topoisomerase IV subunit B |
|  |  | Translation elongation factor Tu |
|  |  | Outer membrane porin OmpF |
|  |  | H repeat-associated protein, YhhI family |
| DOX120 | | DNA-directed RNA polymerase beta subunit |
|  |  | Cyclic AMP receptor protein |
|  |  | Putative esterase YheT functionally coupled to phosphoribulokinase homolog |
|  |  | Aerobic respiration control response regulator ArcA |
|  |  | Multiple antibiotic resistance protein MarR |
|  |  | Serine recombinase, PinQ/PinR-type |
|  |  | Protein RhsB |
|  |  | Phage tail fiber, tail fiber assembly protein Tfa |
|  |  | H repeat-associated protein, YhhI family |

| KAN80 | IS1 protein InsA |
| --- | --- |
|  | CDP-diacylglycerol--glycerol-3-phosphate 3-phosphatidyltransferase |
|  | Cyclic AMP receptor protein |
|  | Putative esterase YheT functionally coupled to phosphoribulokinase homolog |
|  | Translation elongation factor G |
|  | Uncharacterized protein, YnaE family |
|  | Serine recombinase, PinQ/PinR-type |
|  | Translation elongation factor Tu |
|  | Trk potassium uptake system protein TrkA |
|  | H repeat-associated protein, YhhI family |
| NIT30 | Cyclic AMP receptor protein |
|  | Putative esterase YheT functionally coupled to phosphoribulokinase homolog |
|  | Serine recombinase, PinQ/PinR-type |
|  | Translation elongation factor Tu |
|  | Oxygen-insensitive NAD(P)H nitroreductase/ Dihydropteridine reductase |
|  | Multidrug resistance regulator EmrR (MprA) |
|  | Oxygen-insensitive NADPH nitroreductase |
|  | H repeat-associated protein, YhhI family |
| TMP30 | H repeat-associated protein, YhhI family |
|  | Cyclic AMP receptor protein |
|  | Putative esterase YheT functionally coupled to phosphoribulokinase homolog |
|  | Phosphopentomutase |
|  | Serine recombinase, PinQ/PinR-type |
|  | Thymidylate synthase |
|  | Long-chain fatty acid transport protein |
|  | Chromosomal replication initiator protein DnaA |
|  | Isocitrate dehydrogenase [NADP] |

**Table S6. Single Nucleotide Polymorphism (SNPs) of AMPs-evolved strains.**

| **Strains** | **SNPs** |  |
| --- | --- | --- |
| COL120 | IS1 protein InsA |  |
|  | Diguanylate cyclase |  |
|  | Cyclic AMP receptor protein | |
|  | Putative esterase YheT functionally coupled to phosphoribulokinase homolog | |
|  | Uncharacterized protein, YnaE family | |
|  | Serine recombinase, PinQ/PinR-type | |
|  | Translation elongation factor Tu | |
|  | 2-keto-4-pentenoate hydratase | |
|  | H repeat-associated protein, YhhI family | |
|  | Protein RhsB | |
|  | Phage tail fiber, tail fiber assembly protein Tfa | |
|  | H repeat-associated protein, YhhI family | |
| Ind120 | Cyclic AMP receptor protein | |
|  | Putative esterase YheT functionally coupled to phosphoribulokinase homolog | |
|  | Aerobic respiration control response regulator ArcA | |
|  | Uncharacterized protein, YnaE family | |
|  | Serine recombinase, PinQ/PinR-type | |
|  | Translation elongation factor Tu | |
|  | Peptide ABC transporter, permease protein SapC | |
|  | H repeat-associated protein, YhhI family | |

| Omi120 | Cyclic AMP receptor protein |
| --- | --- |
|  | Putative esterase YheT functionally coupled to phosphoribulokinase homolog |
|  | Aerobic respiration control response regulator ArcA |
|  | Serine recombinase, PinQ/PinR-type |
|  | Translation elongation factor Tu |
|  | Ribonuclease PH |
|  | H repeat-associated protein, YhhI family |
| Pex120 | Cyclic AMP receptor protein |
|  | Putative esterase YheT functionally coupled to phosphoribulokinase homolog |
|  | Uncharacterized lipoprotein YsaB precursor |
|  | Uncharacterized protein, YnaE family |
|  | Serine recombinase, PinQ/PinR-type |
|  | Translation elongation factor Tu |
|  | Peptide ABC transporter, ATP-binding protein SapD |
|  | H repeat-associated protein, YhhI family |
|  | Sensor protein BasS/PmrB |
| PGLA120 | Cyclic AMP receptor protein |
|  | Putative esterase YheT functionally coupled to phosphoribulokinase homolog |
|  | Aerobic respiration control response regulator ArcA |
|  | Uncharacterized protein YegL |
|  | Translation elongation factor Tu |
|  | Ribonuclease PH |
|  | H repeat-associated protein, YhhI family |

| Pleu120 | Cyclic AMP receptor protein |
| --- | --- |
|  | Putative esterase YheT functionally coupled to phosphoribulokinase homolog |
|  | Aerobic respiration control response regulator ArcA |
|  | Uncharacterized protein, YnaE family |
|  | Serine recombinase, PinQ/PinR-type |
|  | Protein RhsB |
|  | core protein |
|  | Translation elongation factor Tu |
|  | H repeat-associated protein, YhhI family |
| R8120 | Cyclic AMP receptor protein |
|  | Putative esterase YheT functionally coupled to phosphoribulokinase homolog |
|  | Aerobic respiration control response regulator ArcA |
|  | Uncharacterized protein, YnaE family |
|  | Serine recombinase, PinQ/PinR-type |
|  | Translation elongation factor Tu |
|  | Peptide ABC transporter, permease protein SapC |
|  | H repeat-associated protein, YhhI family |
| SAAP120 | Cyclic AMP receptor protein |
|  | Putative esterase YheT functionally coupled to phosphoribulokinase homolog |
|  | Uncharacterized protein, YnaE family |
|  | RNA polymerase sigma factor RpoS |
|  | Translation elongation factor Tu |
|  | Phage tail fiber, tail fiber assembly protein Tfa |
|  | DNA-directed RNA polymerase alpha subunit |
|  | H repeat-associated protein, YhhI family |

| LI120 | IS1 protein InsA |
| --- | --- |
|  | Cyclic AMP receptor protein |
|  | Putative esterase YheT functionally coupled to phosphoribulokinase homolog |
|  | Uncharacterized protein, YnaE family |
|  | Serine recombinase, PinQ/PinR-type |
|  | RNA polymerase sigma factor RpoS |
|  | Translation elongation factor Tu |
|  | H repeat-associated protein, YhhI family |
| SLAP120 | Cyclic AMP receptor protein |
|  | Putative esterase YheT functionally coupled to phosphoribulokinase homolog |
|  | Aerobic respiration control response regulator ArcA |
|  | Lipopolysaccharide export system protein LptC |
|  | Uncharacterized protein, YnaE family |
|  | Serine recombinase, PinQ/PinR-type |
|  | Protein RhsB |
|  | Translation elongation factor Tu |
|  | Glutamate decarboxylase |
|  | H repeat-associated protein, YhhI family |

**Table S7. MIC values of evolved MG1655 to different antibiotics (μg/mL).**

| Antibiotics | Strains | | | | | | | | |
| --- | --- | --- | --- | --- | --- | --- | --- | --- | --- |
|  | AMP120 | AZI120 | CHL80 | CIP120 | DOX120 | KAN80 | NIT70 | TMP30 | COL120 |
| AMP | 64 | 2 | 64 | 16 | 32 | 4 | 4 | 4 | 4 |
| AZI | 1 | 16 | 2 | 4 | 2 | 2 | 4 | 16 | 0.5 |
| CHL | 16 | 8 | 512 | 32 | 64 | 2 | 8 | 8 | 8 |
| CIP | 0.0078 | 0.0078 | 0.0625 | 2 | 0.0156 | 0.0078 | 0.0078 | 0.00195 | 0.0078 |
| DOX | 2 | 8 | 16 | 16 | 32 | 1 | 8 | 4 | 4 |
| KAN | 1 | 4 | 2 | 4 | 2 | 512 | 4 | 4 | 4 |
| NIT | 16 | 16 | 4 | 16 | 16 | 8 | 512 | 4 | 8 |
| TMP | 128 | 64 | 128 | 128 | 128 | 128 | 128 | 512 | 128 |

**Table S8. MIC values of evolved MG1655 to different AMPs (μg/mL).**

| AMPs | Strains | | | | | | | | |
| --- | --- | --- | --- | --- | --- | --- | --- | --- | --- |
|  | AMP120 | AZI120 | CHL80 | CIP120 | DOX120 | KAN80 | NIT70 | TMP30 | COL120 |
| COL | 0.5 | 0.5 | 0.25 | 0.5 | 0.25 | 0.5 | 0.5 | 0.5 | 0.5 |
| Ind | 16 | 8 | 16 | 16 | 8 | 4 | 8 | 16 | 8 |
| Omi | 8 | 8 | 8 | 8 | 8 | 8 | 8 | 8 | 8 |
| Pex | 2 | 4 | 4 | 2 | 2 | 2 | 4 | 1 | 4 |
| PGLA | 4 | 8 | 4 | 4 | 4 | 4 | 8 | 4 | 8 |
| Pleu | 1 | 1 | 1 | 1 | 1 | 1 | 1 | 1 | 1 |
| R8 | 4 | 8 | 8 | 8 | 8 | 4 | 8 | 8 | 4 |
| SAAP | 16 | 64 | 16 | 32 | 16 | 16 | 32 | 16 | 32 |
| LI | 8 | 8 | 2 | 8 | 4 | 4 | 8 | 8 | 8 |
| SLAP | 1 | 32 | 2 | 2 | 2 | 2 | 2 | 4 | 8 |

**Table S9. MIC values of MG1655 with gene silencing to four drugs (μg/mL).**

| Drugs | Strains | | | | |
| --- | --- | --- | --- | --- | --- |
|  | WT | *dnaA*-i | *deoB*-i | *icd*-i | *thyA*-i |
| TMP | 64 | 64 | 128 | 128 | 256 |
| CIP | 0.0078 | 0.0039 | 0.0078 | 0.0078 | 0.00195 |
| NIT | 16 | 16 | 16 | 16 | 8 |
| Pex | 4 | 2 | 2 | 4 | 1 |

**Table S10. Primer sequences used for plasmids construction and RT-qPCR analysis.**

| Gene | Primer applications | Forward Primer Sequence | Reverse Primer Sequence |
| --- | --- | --- | --- |
| *dnaA* | plasmids construction | CAGGTGGCGGATAACCCTGGGTTTTAGAGCTAGAAATAGC | CCAGGGTTATCCGCCACCTGACTAGTATTATACCTAGGAC |
| *deoB* |  | TGATGAAAAACACGGCCAGGGTTTTAGAGCTAGAAATAGC | CCTGGCCGTGTTTTTCATCAACTAGTATTATACCTAGGAC |
| *icd* |  | GAAGGAGCGTTTAAAGACTGGTTTTAGAGCTAGAAATAGC | CAGTCTTTAAACGCTCCTTCACTAGTATTATACCTAGGAC |
| *thyA* |  | GGCCAGTGTATGGTAAACAGGTTTTAGAGCTAGAAATAGC | CTGTTTACCATACACTGGCCACTAGTATTATACCTAGGAC |
| *dnaA* | RT-qPCR analysis | AAAGTCGCGGATCTCCTTTC | GACTGTGGTTAGTCAGCTCTTT |
| *deoB* |  | GATTGCCTGCCATGAAGAAAC | GAGCGATAACACGACCGATATT |
| *icd* |  | CTGCAGTCGAGAAAGCCTATAA | CCAGACGTCCTGACCATAAAC |
| *thyA* |  | GAACGTAGGCGAACTGGATAAA | GTCACAGGAGCGCTGATAAA |
